# Turmeric active substance, curcumin, enhanced apomorphine-induced yawning in rats

**Published:** 2013

**Authors:** Esmaeal Tamaddonfard

**Affiliations:** 1 *Department of Basic Sciences, Faculty of Veterinary Medicine, Urmia University, Urmia 57153-1177, I.R. Iran*

**Keywords:** Apomorphine, Curcumin, Haloperidol, Rats, Yawning

## Abstract

**Objective: **Curcumin is a major constituent of turmeric and influences many functions of the brain. In the present study, we investigated the effect of curcumin on yawning induced by apomorphine in rats.

**Materials and Methods: **Curcumin administered orally for 10 consecutive days. Yawning was induced by subcutaneous (s.c.) injection of apomorphine (a dopamine receptor agonist) and the number of yawns was recorded for a period of 30 min.

**Results: **Apomorphine (0.05 and 0.1 mg/kg) produced yawning. Haloperidol (a dopamine receptors antagonist) at a dose of 0.05 mg/kg partially and at a dose of 0.2 mg/kg completely inhibited apomorphine-induced yawning. Curcumin alone produced no yawning, whereas at doses of 30 and 60 mg/kg, it increased yawning induced by 0.1 mg/kg of apomorphine. Curcumin at the high doses (30 and 60 mg/kg) produced yawning when apomorphine (0.1 mg/kg) action was partially blocked with 0.5 mg/kg of haloperidol. In the presence of complete blockade of apomorphine (0.1 mg/kg) action with 0.2 mg/kg of haloperidol, curcumin did not produce yawning.

**Conclusion:** The results showed that curcumin at high doses increased apomorphine-induced yawning. In the presence of partial, but not complete blockade of apomorphine action, curcumin produced yawning. Curcumin produced a dopamine-like effect on yawning.

## Introduction

Yawning is a common stereotype behavior that occurs in most vertebrates and humans (Baenninger and Greco, 1991 ▶). In mammals, it consists of an involuntary sequence of mouth opening, deep inspiration, brief apnea, and slow expiration (Baenninger, 1997 ▶). Yawning is under a coordinated control of several neurotransmitters and neuropeptides such as histamine, acetylcholine, excitatory amino acids, γ-aminobutyric acid (GABA), oxytocin, and opioid peptides in the central nervous system (Tamaddonfard et al., 2008a ▶; Collins and Equibar, 2010 ▶). Several lines of pharmacological evidence suggest that dopamine is involved in the yawning expression (Melis et al., 1987 ▶; Zarrindast and Jamshidzadeh, 1992 ▶; Collins et al., 2005 ▶; Li et al., 2010 ▶). 

Curcumin is the active ingredient in the traditional herbal remedy and dietary spice turmeric and has a surprisingly wide range of beneficial properties, including anti-inflammatory, analgesic, antiepileptic, antioxidant, chemopreventive, and chemotherapeutic activities (Hatcher et al., 2008 ▶; Tamaddonfard et al., 2009 ▶, 2010, 2012). Curcumin influences brain neurotransmission, function, and behavior (Kulkarni et al., 2008 ▶; Wang et al., 2008 ▶; Bhutani et al., 2009 ▶). Some evidences suggest protective roles for curcumin in brain pathology. Oral administration of curcumin has resulted in the inhibition of amyloid β deposition and oligomerization, and tau phosphorylation in the brains of Alzheimer disease animal models, and improvements in behavioral impairment in animal models (Hamaguchi et al., 2010 ▶). In intracerebral hemorrhage model in mice, curcumin prompted hematoma resolution and limited neurological injury (King et al., 2011 ▶).

In the present study, we investigated the effects of oral administrations of curcumin on yawning induced by apomorphine. In the presence of partial and complete blockades of apomorphine action with low and high doses of haloperidol, the effects of curcumin on yawning were also investigated. 

## Materials and Methods

**Animals**

Healthy adult male Wistar rats, weighing 240–250 g were used in this study. Rats were provided from rat house of laboratory of physiology of Veterinary Medicine Faculty of Urmia University. Animals were maintained in polyethylene cages with food and water available *ad libitum* in a laboratory with controlled ambient temperature (22±0.5 °C) and under a 12 h light-dark cycle (lights on 07:00 h). Experiments were carried out between 13:00 h and 17:00 h. Eight rats were used in each experiment. The experimental protocol was approved by the Veterinary Ethics Committee of the Faculty of Veterinary Medicine of Urmia University and was performed in accordance with the National Institutes of Health Guide for Care and Use of Laboratory Animals.

**Drugs and Chemicals**

Curcumin, apomorphine, and haloperidol were purchased from Sigma–Aldrich Co., St Louis, MO, USA. Apomorphine and haloperidol were dissolved in sterile normal saline. A suspension of curcumin was provided in sterile normal saline. 

**Treatment groups**

The rats were divided into the following groups: normal saline, apomorphine, haloperidol, haloperidol plus apomorphine, curcumin, curcumin plus apomorphine, and curcumin plus haloperidol plus apomorphine groups. Apomorphine at doses of 0.05 and 0.1 mg/kg, haloperidol at doses of 0.05 and 0.2 mg/kg alone and prior to apomorphine (0.1 mg/kg) were (s.c.) injected. Curcumin at doses of 3.75, 6.5, 15, 30, and 60 mg/kg were orally administered for 10 consecutive days. All doses of curcumin were examined with apomorphine (0.1 mg/kg). 

The effects of curcumin on yawning were evaluated with partial (0.05 mg/kg haloperidol plus 0.1 mg/kg of apomorphine) and complete (0.2 mg/kg haloperidol plus 0.1 mg/kg of apomorphine) blockades of dopaminergic system. 

Curcumin suspension in normal saline was freshly prepared and administered orally. Yawning recording was began 20 and 10 min after (s.c.) injections of haloperidol and apomorphine, respectively, and 1 h after last oral administration of curcumin. In the present study, doses of used drugs were selected on the basis of literature, that is, 3.125-25 mg/kg for long (15 days) use of curcumin (Tamaddonfard et al., 2010 ▶), 0.001-0.32 mg/kg for apomorphine (Collins et al., 2005, 2007), and 0.01-0.1 mg/kg for haloperidol (Collins et al., 2005 ▶, 2007). 

**Induction of yawning**

Yawning behavior was defined as a prolonged (more than 1 s) wide opening of the mouth followed by a rapid closure (Collins et al., 2005 ▶, 2007; Tamaddonfard et al., 2008a ▶). On the day of testing, rats were placed in plexiglass chambers (30**×**25**×**25 cm) and allowed to adapt to the chamber for a period of 30 min. Blinded observers recorded the number of yawns after application of drugs.

**Statistical analyses**

Data were analyzed by one-way analysis of variance (ANOVA) and Duncan’s test. All the values are expressed as the mean±SEM. Statistical significance was set at p<0.05.

## Results

**Figure 1:**
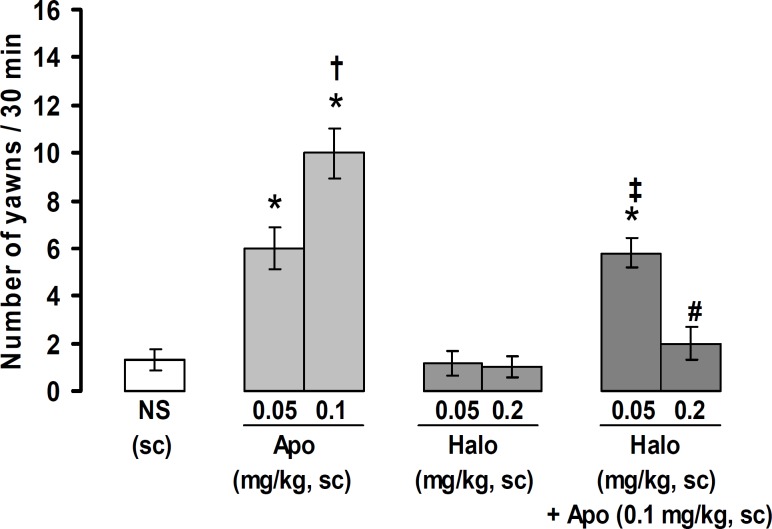
The number of yawns produced by apomorphine and haloperidole and the effect of haloperidol on apomorphine-induced yawning. All values are expressed as mean±SEM (n=8). *p<0.05 in comparison with normal saline, ^†^p<0.05 in comparison with apomorphine (0.05 mg/kg), ^‡^p<0.05 in comparison with apomorphine (0.1 mg/kg), ^#^p<0.05 in comparison with apomorphine (0.05 and 0.1 mg/kg) and haloperidol (0.05 mg/kg) + apomorphine (0.1 mg/kg). NS: normal saline, Apo: apomorphine, Halo: haloperidol, sc: subcutaneous

Figure 1 shows the number of yawns induced by injections of apomorphine, haloperidol, and haloperidol plus apomorphine. The number of yawns produced by (s.c.) injection of apomorphine (0.1 mg/kg) showed significant differences with those obtained from (s.c.) injection of apomorphine 0.05 mg/kg and normal saline. A significant difference was observed between apomorphine 0.05 mg/kg and normal saline (p<0.05). No significant differences were observed between normal saline and 0.05 and 0.2 mg/kg of haloperidol. The inhibitory effect of haloperidol (0.2 mg/kg) was significantly more potent than that obtained from haloperidol (0.5 mg/kg) on apomorphine (0.1 mg/kg)-induced yawning (p<0.05). 

**Figure 2:**
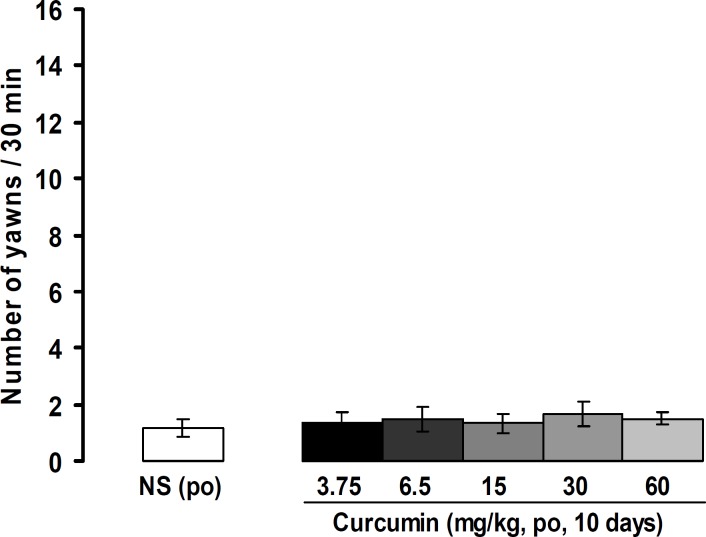
Yawning responses induced by oral administration of normal saline and curcumin. All values are expressed as the mean±SEM (n=8). Apo: apomorphine, Halo: haloperidol, po: oral administration

Figure 2 shows the number of yawns induced by oral administrations of normal saline and curcumin. Curcumin produced a negligible yawning response that showed no significant difference with normal saline. 

**Figure 3:**
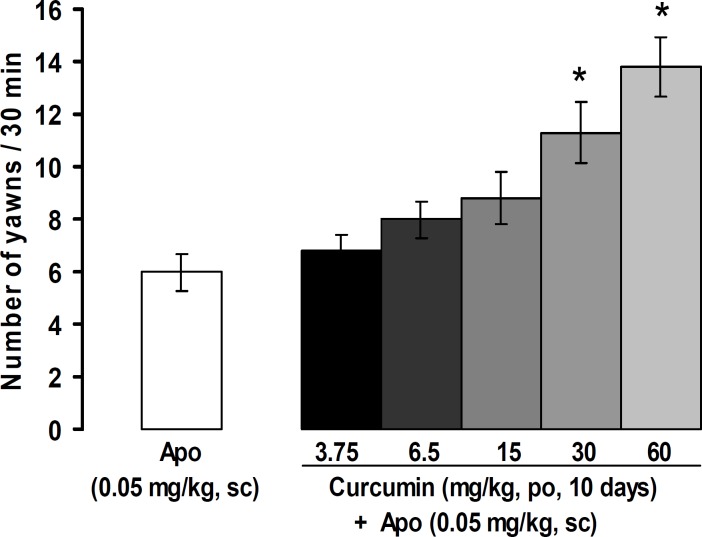
Effects of oral administration of curcumin on yawning induced by apomorphine. All values are expressed as the mean±SEM (n=8). *p<0.05 in comparison with apomorphine (0.05 mg/kg). NS: normal saline, Apo: apomorphine, Halo: haloperidol, sc: subcutaneous, po: oral administration

Figure 3 shows the effects of oral administrations of curcumin on apomorphine-induced yawning. Oral administrations of curcumin at doses of 3.75, 6.5, and 15 mg/kg did not change the number of yawns, whereas at doses of 30 and 60 mg/kg, curcumin significantly increased apomorphine (0.05 mg/kg)-induced yawning (p<0.05). 

**Figure 4:**
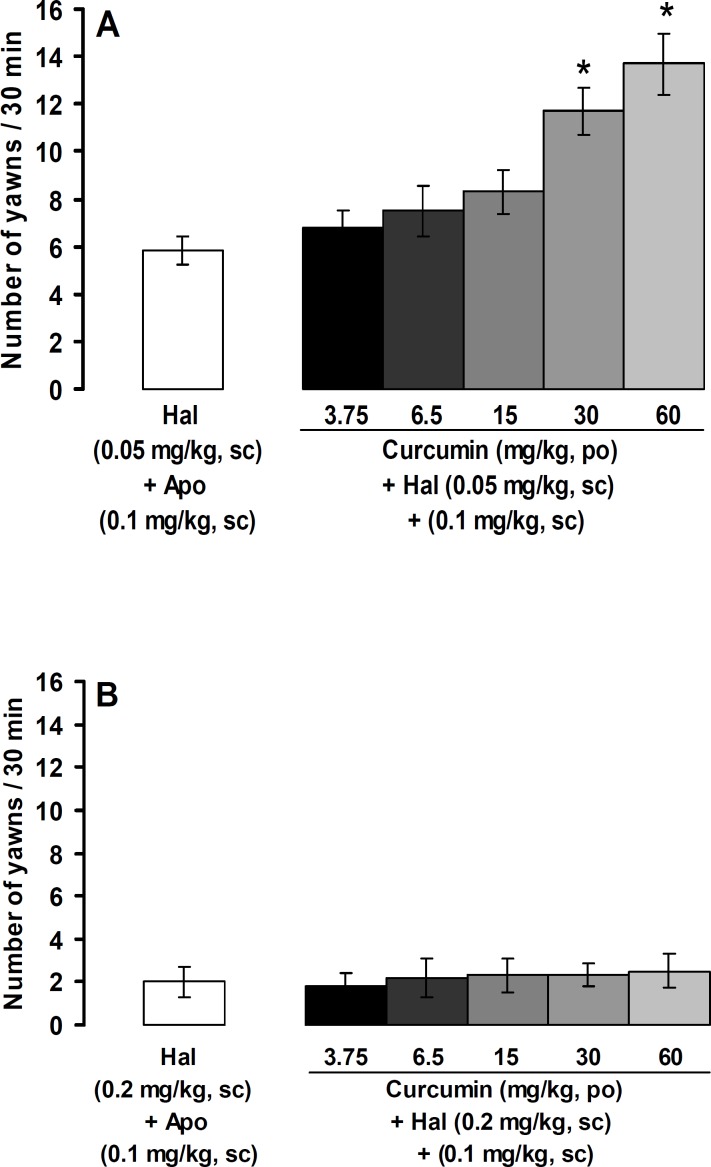
Yawning responses induced by curcumin in the presence of partial (A) and complete (B) blockades of apomorphine action with haloperidol. All values are expressed as the mean±SEM (n=8). *p<0.05 in comparison with haloperidol (0.05 mg/kg) + apomorphine (0.1 mg/kg). NS: normal saline, Apo: apomorphine, Halo: haloperidol, sc: subcutaneous, po: oral administration

Figure 4 shows the effects of curcumin on the number of yawns in the presence of partial (Figure 4A) and complete (Figure 4B) blockades of apomorphine action with haloperidol. Oral administration of curcumin at doses of 30 and 60 mg/kg, but not at doses of 3.75, 6.5, and 15 mg/kg, produced yawning in the presence of partial blockade of apomorphine (0.1 mg/kg)-induced yawning with 0.05 mg/kg of haloperidol (p<0.05, Figure 4A). In the presence of complete blockade of apomorphine (0.1 mg/kg)-induced yawning with haloperidol (0.2 mg/kg), oral administrations of curcumin at all used doses did not produce yawning (Figure 4B). 

## Discussion

In the present study, apomorphine produced yawning and haloperidol inhibited apomorphine-induced yawning. This confirms the dopaminergic system involvement in yawning phenomena. The involvement of dopamine in yawning was first suggested by the discovery that dopamine receptor agonists such as apomorphine, bromocriptine, and lisuride were able to induce yawning in rats (Baggio and Ferrari, 1983 ▶; Melis et al., 1987 ▶; Ushijima et al., 1988 ▶). Using dopamine agonists such as apomorphine, bromocriptine, pramipexole, quinelorane, and quinpirol, and dopamine antagonists including L-721 626, U991194, and nafadotride, the involvement of dopaminergic system in yawning was firmly confirmed (Collins et al., 2005 ▶, 2007). Haloperidol, a nonselective dopaminergic antagonist with high affinity for all dopamine receptor subtypes (Sokoloff et al., 1992 ▶), inhibited PD-128907-, quinelorane-, and apomorphine-induced yawning in rats (Collins et al., 2005 ▶, 2007). 

In the present study, we used oral administration of a normal saline suspension of curcumin. Curcumin is insoluble in water, but is soluble in ethanol, alkalis, ketone, acetic acid, and chloroform (Araujo and Leon, 2001 ▶). Oral administrations of a normal saline suspension of curcumin have been frequently used to survey its biological and pharmacological properties (Tamaddonfard et al., 2008b ▶, 2009; Buadonpri et al., 2009 ▶). We observed curcumin responses with high (30 and 60 mg/kg), but not low and medium (3.75, 6.5, and 15 mg/kg) doses. This result is in agreement with other findings in which responses to high doses of curcumin were studied (Tamaddonfard et al., 2008, 2009; Buadonpri et al., 2009 ▶). This effect of curcumin may partly be due to its low systemic bioavailability following oral administration due to efficient first-pass metabolism and some degrees of intestinal metabolism (Sharma et al., 2007 ▶). Although curcumin has low systemic bioavailability after oral administration, the achievement of efficacious concentrations in the gastrointestinal tract is sufficient for exerting beneficial effects in animals and humans (Sharmaet al., 2005 ▶).

In the present study, curcumin increased apomorphine-induced yawning. In addition, in the presence of partial blockade of apomorphine action with haloperidol, curcumin produced yawning. Moreover, when apomorphine effect was completely blocked with haloperidol, curcumin did not produce yawning. These indicate that curcumin contributes to dopaminergic system in producing yawning. Curcumin exhibits antioxidant, anti-inflammatory, and anti-cancer properties, crosses the blood-brain barrier and is neuroprotective in neurological disorders such as Parkinson̕ s disease (Mythri and Bharat, 2012 ▶). Curcumin potentially affects dopaminergic neuron function and health in the brain. Acute administration of curcumin at high (40 and 80 mg/kg), but not at low (10 and 20 mg/kg) doses, increased dopamine levels in the brain (Kulkarni et al., 2008 ▶).

Curcumin pretreatment mitigated lipopolysaccharide-induced dopaminergic neuron degeneration and curcumin post-treatment showed protective effects (Yang et al., 2008 ▶). Long-term (21 days) administration of haloperidol increased vacuous chewing movements and facial jerkings and decreased turnover of dopamine in cortical and subcortical regions of the brain. Chronic pretreatment with curcumin reversed all changes induced by haloperidol (Bishoni et al., 2008 ▶). In addition, chronic oral administration of curcuminoids including curcumin, demethoxycurcumin, and bisdemethoxycurcumin prevented striatal dopaminergic neurodegeneration induced by 6-hydroxydopamine model of Parkinsonism in rats (Agrawal et al., 2012 ▶). Curcumin, through its iron-chelating property, produced protective effects on 6-hydroxydopamine-induced degeneration of nigral dopaminergic neurons (Du et al., 2012 ▶). Moreover, curcumin supplementation reduced alternation of dopamine D_1_ and D_2_ receptors in cerebral cortex and cerebellum of diabetic rats (Kumar et al., 2010 ▶). It seems that curcumin can affect brain neurotransmitter function through multiple pathways including gene expression, receptor protection, and neurotransmitter level changing. 

In conclusion, the results of the present study indicated that apomorphine by activating endogenous dopamine produced yawning, and haloperidol, a dopaminergic antagonist, blocked apomorphine-induced yawning. Curcumin increased the yawning induced by apomorphine. In the presence of partial, but not complete blockade of apomorphine action with haloperidol, curcumin produced yawning. Curcumin, at least, in the present study exerted a dopamine-like activity. 
